# Correction: Axial variation of deoxyhemoglobin density as a source of the low-frequency time lag structure in blood oxygenation level-dependent signals

**DOI:** 10.1371/journal.pone.0225489

**Published:** 2019-11-14

**Authors:** Toshihiko Aso, Shinnichi Urayama, Hidenao Fukuyama, Toshiya Murai

In the “Source of BOLD low-frequency oscillation signals” subsection of the Discussion, the second and third sentences of the fourth paragraph should be omitted. The correct paragraph is: In the hyperventilation condition, we observed a fast response to each ventilation cycle, accompanying blood pressure changes. Although the effect of respiratory movement cannot be fully excluded, the spatial pattern in S2B Fig is not that of a typical motion artifact centered on the brain surface [92]. In healthy participants, inhalation increases systemic venous return through decreased intrathoracic pressure, causing an elevation of cardiac output with some delay. In contrast, exhalation is considered to cause CBV increases through elevated cerebral venous pressure. To our knowledge, the timing order of these events has not been studied at the precision of the current data; further studies are needed to determine the source of this S_0_ fluctuation [59]. The only available clue in our results is the spatial distribution, such as the interesting anterior-posterior segmentation (resembling the unique “S_0_ lag structure” in Fig 9A) or the symmetrical pattern in the deep brain structures. Nonetheless, some mechanical effects of the respiratory act on the fluid dynamics likely exist, causing this spatially heterogeneous S_0_ deflection.

As a result of the correction to the Discussion, References 88–91 are no longer cited in the article.
