# Supplementary figures and images for: Axial variation of deoxyhemoglobin density as a source of the low-frequency time lag structure in blood oxygenation level-dependent signals
Source: PLoS One. 2019 Sep 23;14(9):e0222787. doi: 10.1371/journal.pone.0222787 (PMC6756514; doi:10.1371/journal.pone.0222787)

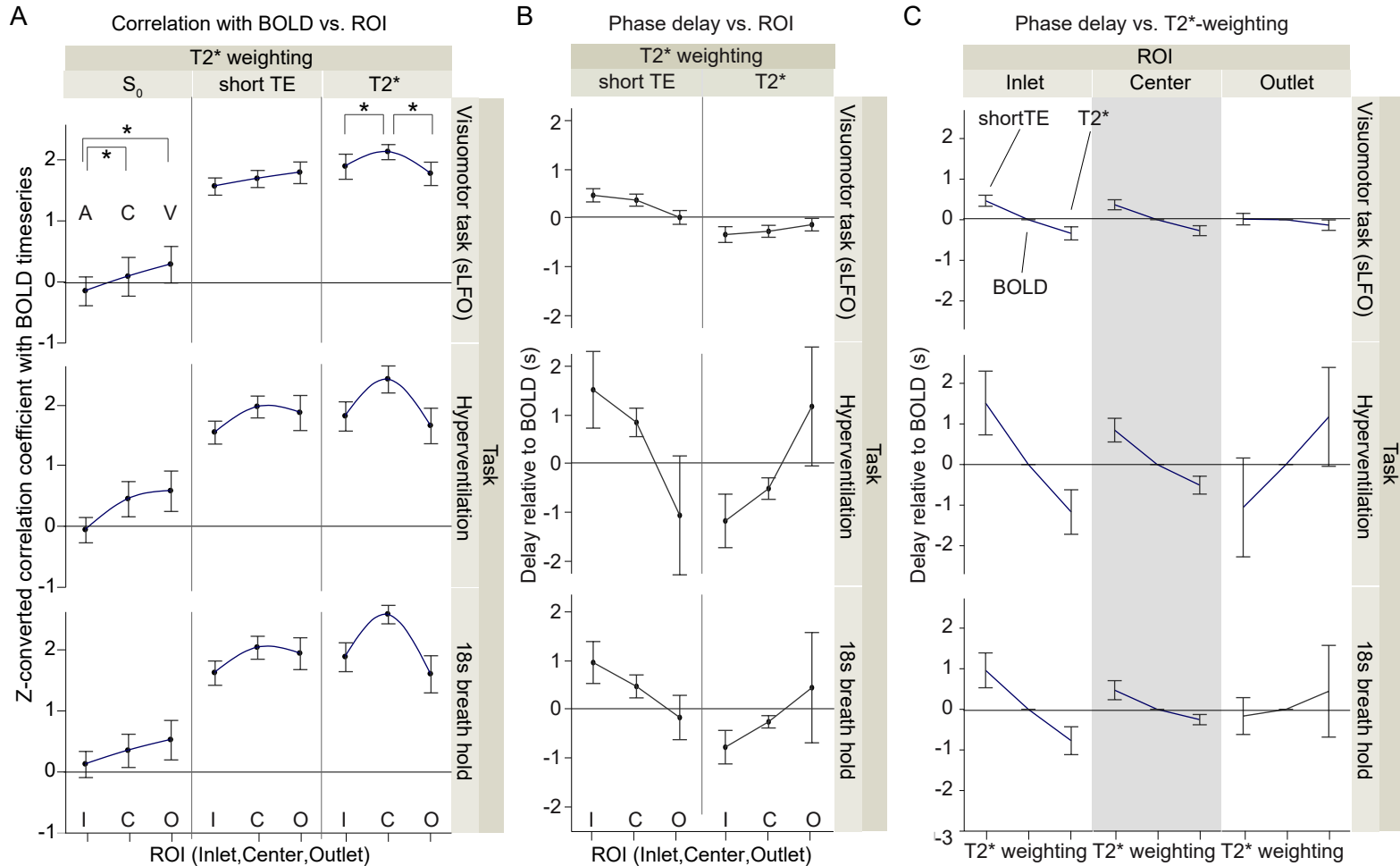

Supplement: S1 Fig — A, Correlation analysis of the multi echo-derived signals extracted from the three vascular regions. Effects of vascular region was observed on both S0 and T2* components, but in a different manner. B, Phase analysis of the low-frequency component below 0.1 Hz. Phase delay relative to BOLD time course was calculated for the two T2* weighted signals. Respiratory challenges enhanced the phase difference between S0 and T2*. C. The same data as in B, but separately plotted for each region. Dissociation of the T2* and S0 phase, as well as its interaction with the vascular region is evident. All these effects were small with spontaneous low-frequency fluctuation but enhanced in artificial oscillation by respiratory challenges. (PDF) [file pone.0222787.s001.pdf]
