# Supplementary material for: Axial variation of deoxyhemoglobin density as a source of the low-frequency time lag structure in blood oxygenation level-dependent signals
Source: PLoS One. 2019 Sep 23;14(9):e0222787. doi: 10.1371/journal.pone.0222787 (PMC6756514; doi:10.1371/journal.pone.0222787)
Supplement: S2 Fig — A, Raw signal responses in the motor/premotor activation cluster for each task from Experiment 2. Time courses were resampled to a sampling interval of 0.5 s. Small but distinct responses were found in the non-BOLD or S0 component but with a dominating fast component compared to the regional response, apparently independent of both the neurovascular coupling and hemoglobin fluctuations that accompany T2* changes. This response was roughly out of phase with the beat-to-beat mean arterial blood pressure shown in Fig 3, although the blood pressure response was absent during the first two cycles. Arrows indicate the fluctuation corresponding to the neurovascular coupling modelled in the SPM analysis. B. Group SPM to assess the distribution of the respiratory phase-related fast fluctuation of S0 during hyperventilation. A separate SPM analysis was performed by modeling the fast S0 fluctuation only. An anterior-posterior segmentation is evident. In addition, there are small symmetrical structures along the major veins. C. Additional experiment from a subset of participants (N = 7) using a different set of repetition time/flip angle to manipulate the T1-inflow effect on the S0 component. The overall S0 response is lower than those shown in panel A, but the fast response to hyperventilation is preserved. (PDF) [file pone.0222787.s002.pdf]

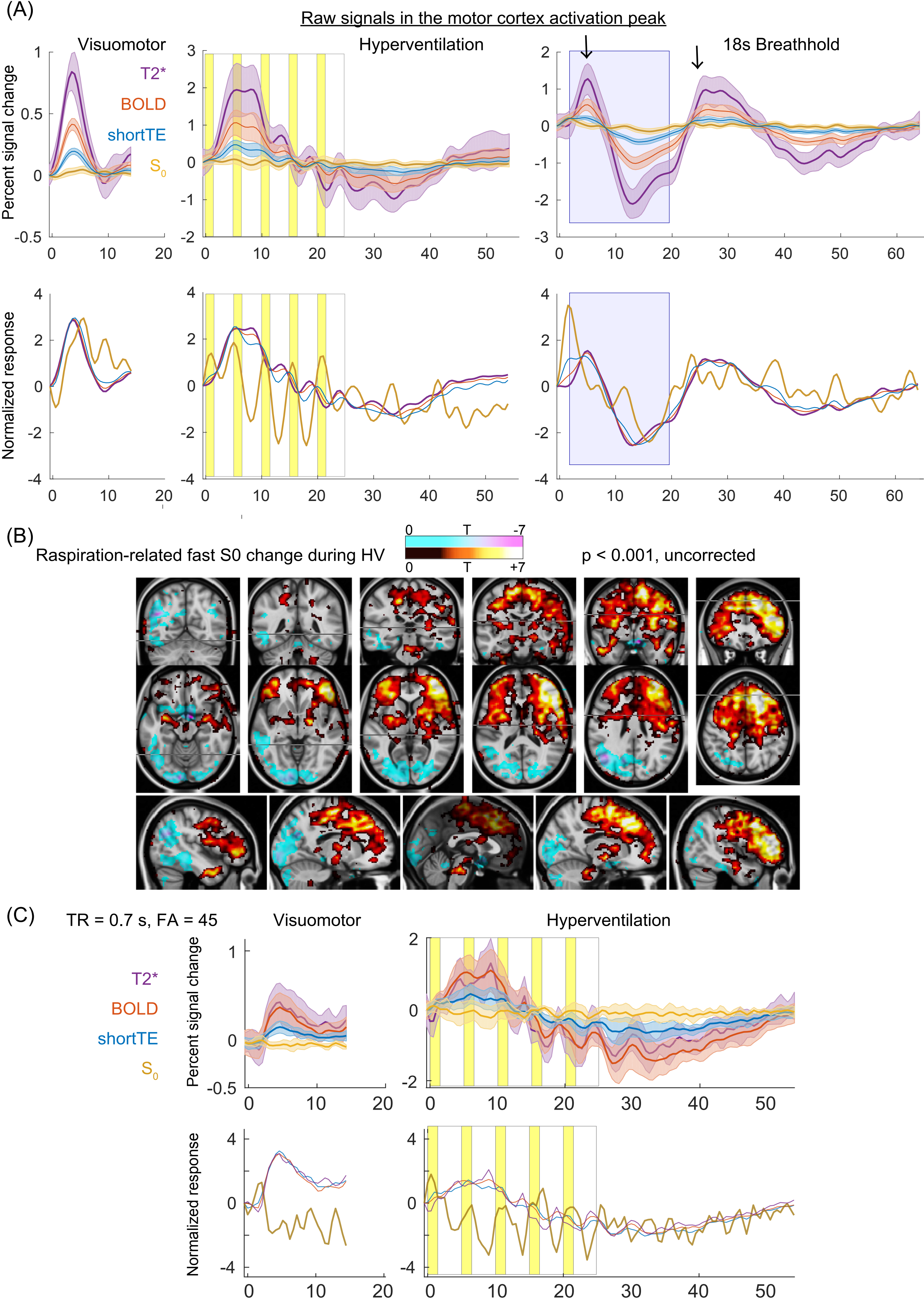
(A)
Raw signals in the motor cortex activation peak
Visuomotor
Hyperventilation
18s Breathhold
T2*
BOLD
shortTE
S0
Percent signal change
Normalized response
(B)
Raspiration-related fast S0 change during HV
p < 0.001, uncorrected
0
T
-7
0
T
+7
(C)
TR = 0.7 s, FA = 45
Visuomotor
Hyperventilation
T2*
BOLD
shortTE
S0
Percent signal change
Normalized response
